# Nonunique UPGMA clusterings of microsatellite markers

**DOI:** 10.1093/bib/bbac312

**Published:** 2022-08-01

**Authors:** Natàlia Segura-Alabart, Francesc Serratosa, Sergio Gómez, Alberto Fernández

**Affiliations:** Departament d’Enginyeria Informática i Matemátiques, Universitat Rovira i Virgili, Av. Països Catalans 26, 43007, Tarragona, Spain; Departament d’Enginyeria Informática i Matemátiques, Universitat Rovira i Virgili, Av. Països Catalans 26, 43007, Tarragona, Spain; Departament d’Enginyeria Informática i Matemátiques, Universitat Rovira i Virgili, Av. Països Catalans 26, 43007, Tarragona, Spain; Departament d’Enginyeria Quámica, Universitat Rovira i Virgili, Av. Països Catalans 26, 43007, Tarragona, Spain

**Keywords:** microsatellite marker, SSR, STR, dendrogram, UPGMA, tie in proximity

## Abstract

Agglomerative hierarchical clustering has become a common tool for the analysis and visualization of data, thus being present in a large amount of scientific research and predating all areas of bioinformatics and computational biology. In this work, we focus on a critical problem, the nonuniqueness of the clustering when there are tied distances, for which several solutions exist but are not implemented in most hierarchical clustering packages. We analyze the magnitude of this problem in one particular setting: the clustering of microsatellite markers using the Unweighted Pair-Group Method with Arithmetic Mean. To do so, we have calculated the fraction of publications at the Scopus database in which more than one hierarchical clustering is possible, showing that about 46% of the articles are affected. Additionally, to show the problem from a practical point of view, we selected two opposite examples of articles that have multiple solutions: one with two possible dendrograms, and the other with more than 2.5 million different possible hierarchical clusterings.

## Introduction

Molecular markers are powerful tools to study genetic diversity. They can be used to identify and characterize the genetic variation (different genotypes) within and between species and populations [1]. Numerous molecular genetic markers are available for genetic variation studies: isozyme, directed amplification of minisatellite DNA, random amplified polymorphic DNA, amplified fragment length polymorphism, inter-simple sequence repeat, restriction fragment length polymorphism and microsatellite markers [1–3]. Among the different molecular markers, microsatellites, which are also known as short tandem repeats (STR) or simple sequence repeats (SSR), show the following advantages: they are highly reproducible; they are co-dominant and multiallelic; they are highly polymorphic, thus allowing precise discrimination between closely related genotypes and they can be analyzed by a polymerase chain reaction assay [4, 5].

Microsatellite markers are short fragments of DNA, between 1 and 6 base pairs repeated in tandem and randomly inside the genome [6]. They have been used for clustering tasks, mainly in the Eukaryota domain, from animals [7, 8] to plants [9] and fungi [10], and in the bacteria domain [11]. For any of these clustering tasks, hierarchical clustering methods are frequently used. These clustering methods seek to create a hierarchy of clusters based on specific features [12]. The graphical structure representation of hierarchical clustering algorithms is a rooted tree called dendrogram. Hierarchical clustering algorithms are classified by the method used to form the final dendrogram. It can either be from small to large clusters (also known as bottom-up or agglomerative clustering) or from large to small clusters (also known as top-down or divisive clustering). The Unweighted Pair-Group Method with Arithmetic Mean (UPGMA) is an agglomerative hierarchical clustering method very used in practice. It combines step-by-step the nearest two clusters or elements into a higher-level cluster, and the distance between the new cluster and any other cluster is calculated as the arithmetic mean distance between elements in different clusters [13, 14].

All agglomerative hierarchical clustering methods start from a proximity matrix of either similarities or distances between elements [15]. In the case of microsatellite markers, the similarity between any two genotypes is measured as the proportion of shared alleles. Software packages frequently use two alternative distances: one minus the proportion of shared alleles; or minus the logarithm of the proportion of shared alleles. Since the number of shared alleles can only take values between zero and the total number of alleles, and this number of alleles is usually relatively small (e.g. there are 30 microsatellite loci in the first case study analyzed below); thus, it is common to have the same distance value for different pairs of genotypes. Additional ties may also appear at any step of the hierarchical clustering process, and it is even possible to have ties due to the limited resolution (number of decimal digits) used to store the proximities matrix. Therefore, we conclude that the clustering of microsatellite markers is prone to generate tied distances, similarly to what happens in other cases [16].

**Figure 1:**
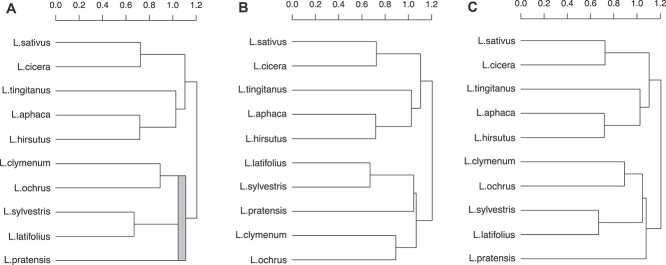
Dendrogram of genetic distances between 10 individuals of *L. sativus* [20]. (**A**) Multidendrogram created with the *mdendro* package, showing in gray the tied cluster grouping *L. clymenum*, *L. ochrus*, *L. sylvestris*, *L. latifolius* and *L. pratensis*, with a range band between the minimum distance (1.0459 units) and the maximum distance (1.1112 units) between all elements that compose the tie in proximity. (**B, C**) The two possible binary dendrograms, where the last five elements are grouped differently.

When there are identical similarity values between different pairs, either in the original distances or during the agglomeration process, agglomerative hierarchical clustering methods in general and UPGMA in particular, can generate more than one structurally different hierarchical clusterings. This condition is known as the ties in proximity problem [13, 16–18]. In all these cases in which multiple clusterings are possible, the reproducibility of the results is more difficult and their interpretation may be biased [19]. In fact, any conclusion obtained from a single binary dendrogram has to be considered partial and, therefore, questionable. For instance, more than one hierarchical clustering is possible when the same distance separates genotype *A* from genotype *B*, as well as genotype *B* from genotype *C*; in this case, genotype *B* can cluster with either genotype *A* or genotype *C*. Figure 1 shows an example extracted from [20]. This example is later explained and analyzed in the Case studies section.

There are several software options to perform hierarchical clustering, developed in different programming environments. For instance, in R, there are the *hclust* (hierarchical clustering) function from the *stats* package [21] and the *agnes* (agglomerative nesting) function from the *cluster* package [22]. In Python, there are the *AgglomerativeClustering* class from the *scikit-learn* package [23], and the *linkage* function from the *scipy* package [24]. And in MATLAB, there is the *linkage* function [25]. All these functions do not try to solve the ties in proximity problem, thus simply returning one of the possible binary dendrograms.

Fortunately, there exist different solutions to properly deal with the ties in proximity problem. One possibility is to measure the likelihood of clusters counting cluster frequencies in the set of all possible binary dendrograms resulting from ties [16]. Another option is to explore all the binary solutions and assess the distances among elements within them [26]. An alternative approach is pyramidal clustering, which allows cluster overlapping to obtain a unique solution [27–29]. And one can also use a variable-group algorithm for agglomerative hierarchical clustering that yields a graphical representation known as multidendrogram, where more than two elements or clusters can be grouped when ties occur [30] (Figure 1A).

In this work, we take a sample of scientific publications that had used the UPGMA method in phylogenetic studies on molecular markers. We analyze the data in these publications using multidendrograms to detect tied distances and count the number of articles where more than one hierarchical clustering was possible. The main purpose of this work is to estimate the proportion of nonunique microsatellite UPGMA trees published in the literature. In addition, to show this issue from a practical point of view, we describe two opposite examples of articles that have more than one possible hierarchical clustering.

## Materials and methods

We carried out the analysis of the articles in three steps. First, we set the search strategy by obtaining the population dataset of articles that used the UPGMA clustering method to classify microsatellite markers. Then, we selected a sample dataset by filtering and analyzing a subset of the publications searching for specific features in them. Finally, we checked whether the selected articles had ties in their hierarchical clusterings and counted the number of articles where the possible hierarchical clusterings were nonunique.

### Search strategy

We looked for scientific publications that used the UPGMA method on microsatellite markers, up to 2021, in the Scopus database. The search query used to retrieve the titles of articles was: ‘UPGMA’ AND (‘microsatellite^*^’ OR ‘simple sequence repeat^*^’ OR ‘SSR’ OR ‘short tandem repeat^*^’ OR ‘STR’), where AND and OR are the standard boolean operators. We added the symbol ^*^ to some words to include the plural form of these words. We limited the search to words of the query present in the title, abstract or keywords, and the publication year up to 2021. We collected the following bibliometric information: document title, journal and year of publication. A total of 2255 articles had been published from 1995 to 2021 (27 years) and a total of 2239 articles remained after removing 16 duplicated records. That was the population dataset subject of this study.

**Figure 2:**
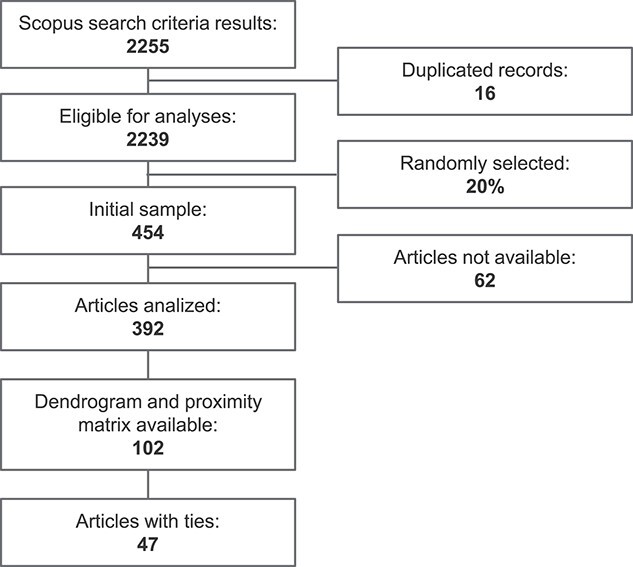
Flowchart of the elaboration of the dataset.

### Sample dataset

Figure 2 shows the flowchart of the dataset. We downloaded all bibliometric information corresponding to the selected articles and randomized the dataset to prevent a bias towards a specific year, subject area or alphabet order. Given the large number of publications included in the dataset ($n$ = 2239), we selected a subset containing 20% of them. As a result, the initial sample dataset was composed of 454 articles. We excluded 62 articles not available. The remaining subset was composed of 392 publications to analyze. From this subset, we only selected the articles that contain a dendrogram and a matrix of proximity data, either similarities or distances, or a table describing the genetic profiles of all genotypes. In the cases where the table with the genetic profiles was provided, we computed a matrix with the proportion of shared alleles using the *adegenet* package [31, 32] in R version 4.1.0 [33]. We rejected articles for further analysis if the proximity data matrix and the dendrogram did not contain the same genotype information. In the end, we came up with a final sample dataset containing 102 articles (see Supplementary data for the complete list).

### Nonunique hierarchical clusterings

We used the *mdendro* package in R to analyze the existence of ties in proximity and to create the corresponding multidendrograms [30, 34]. This package shows the location of any tie in a multidendrogram as a coloured rectangle that represents the variability or range between the minimum and the maximum distances separating any two of the constituent clusters, since it is possible that not all elements in a tie are separated by the same distance (Figure 1A). We also used *Radatools 5.2* [35] to count the number of possible binary hierarchical clusterings corresponding to a given matrix of proximity data. *Radatools* has the option of computing all possible binary dendrograms as well as the unique multidendrogram. We chose the former option as we wanted to calculate the number of binary dendrograms a specific article can have when there are tied clusters.

## Results and discussion

### Proportion of articles with ties in proximity

To count the number of publications that had at least one tie in the resulting hierarchical clustering, we took the proximity data from all the articles in our sample dataset and computed the corresponding multidendrogram. We found that in 47 out of the 102 articles analyzed there was more than one possible binary dendrogram. This value corresponds to 46% of the articles, with a 95% confidence interval (CI) between 36% and 56%. Extrapolating this percentage to the total population of 2239 articles gives an estimate of 1032 articles (95% CI 816–1248 articles) with alternative solutions in the form of different binary dendrograms. In such cases, employing a single arbitrary resolved hierarchical clustering out of the different possibilities can be misleading.

**Figure 3:**
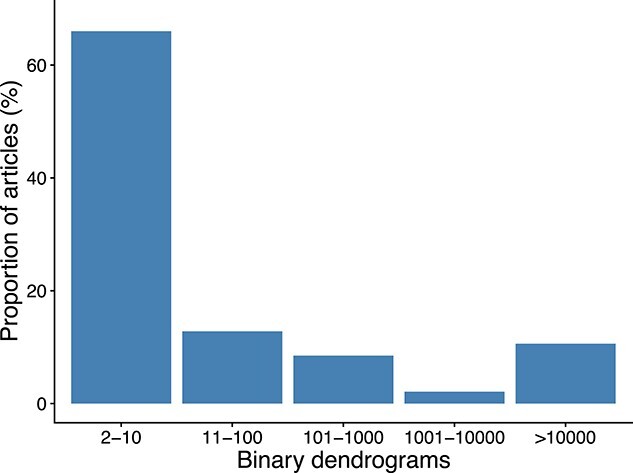
Distribution of the number of binary dendrograms resulted from the articles that had at least one tie in the resulting hierarchical clustering ($n$ = 47).

We were also interested in exploring the distribution of the number of binary dendrograms resulting from the articles that had at least one tie in the resulting hierarchical clustering, see Figure 3. Most articles with ties had between 2 and 10 different binary dendrograms (66%, i.e. 31 of all the articles with ties), followed by articles having between 11 and 100 different binary dendrograms (13%, i.e. six of all the articles with ties). Remarkably, 11% of all the articles with ties (i.e. five articles) had more than 10000 different binary dendrograms. These results are in good agreement with previous studies reporting that the occurrence of ties was responsible for more than one hundred thousand dendrograms [16], or even more than seven hundred million dendrograms [36].

### Analysis of publications per year

**Figure 4:**
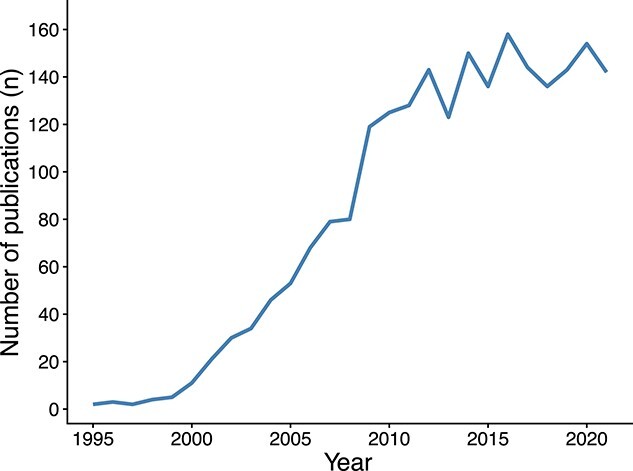
Number of scientific publications that used the UPGMA method on microsatellite markers, up to 2021, in the Scopus database.

The publication year of the articles in our population dataset ranged from 1995 to 2021 (Figure 4). The majority of them were published after year 2000. Since 2009, more than 100 articles have been published yearly; 2016 is the year with more published articles ($n$ = 158) and for the last 10 years the number of publications has stabilized around 140 articles per year. Overall, the number of published articles shows a steady increase since the 2000s, indicating that this research area started to gain considerable attention. The reason for this increase may be 2-fold: on the one side, the existence of next-generation sequencing technologies that started a new era of genomics research with high throughput sequencing data and cheaper sequencing costs [37], and on the other side, software packages to run hierarchical clustering algorithms in general, and the UPGMA method in particular, started to be more readily available at that time.

We are aware that this is just an underestimation of the real number of publications that contain an UPGMA tree of microsatellite markers. This is so because we did not take into consideration articles published in journals outside the Scopus Indexed Journal List. Also, because there are other articles in the Scopus database that contain an UPGMA tree of microsatellite markers, but they do not contain in their title, abstract or keywords, any of the words that we used as search criteria.

### Distribution of subject areas

**Figure 5:**
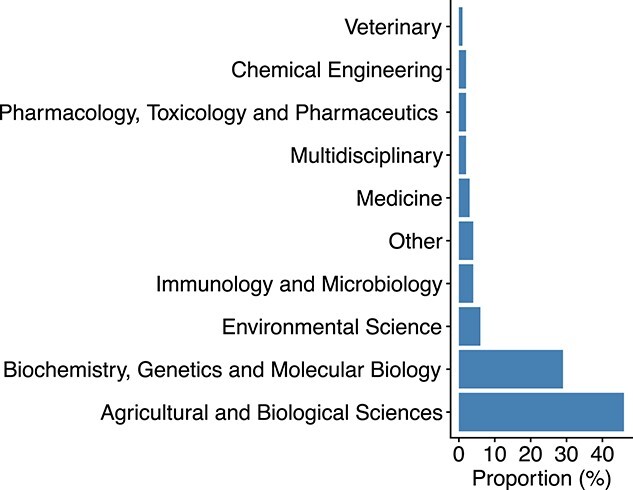
Articles classified by subject area. There are 13 subject areas that constitute <1% each, and they have been grouped together in the category named ‘Other’.

The 2239 articles were classified into 22 different subject areas. The two most common subject areas were Agricultural and Biological Sciences (46%), followed by Biochemistry, Genetics and Molecular Biology (29%) in second place (Figure 5). These two subjects constitute 75% of the total number of articles. It was expected that most of the articles were related to biological sciences or similar research areas as STR and SSR are tools frequently used in these areas. We grouped the 13 subject areas that constitute <1% of the total number of articles each into a category named ‘Other’ in Figure 5. For instance, Computer Science [38], Mathematics [39] or Social Sciences [40] are examples of research areas that are quite distinct from the previous ones. Such a variety of subject areas indicates that the clustering of microsatellite markers by UPGMA is widely used in many areas of scientific knowledge.

**Figure 6:**
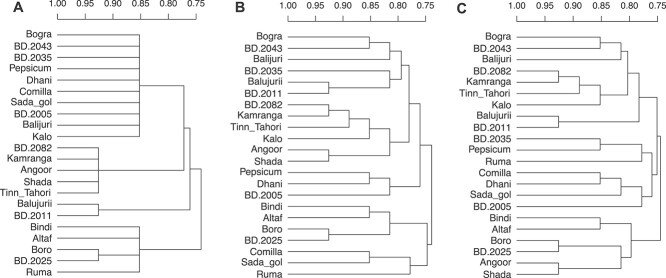
Dendrogram of genetic similarity between 22 individuals of *C. annuum L.* [41]. (**A**) Multidendrogram showcasing the three different ties as a line joining more than two clusters, instead of a range band, for the sake of clarity. (**B, C**) Two possible binary dendrograms among the more than 2.5 million available.

### Case studies

Among the 102 articles that we have analyzed from our sample dataset, we have selected two opposite cases to demonstrate how it is possible to obtain multiple different hierarchical clusterings from the same dataset using the same clustering algorithm (UPGMA). The first example describes a case that generates 2 different binary dendrograms, whereas the second example describes a case that generates more than 2.5 million different binary dendrograms.

In the first case study, the authors analyze the genetic diversity among *Lathyrus sativus* (grasspea) from its cultivated and wild relatives [20]. The study has a total of 10 taxa, the number of microsatellite loci used is 30, and the distance matrix values have an accuracy of four decimal digits. The distance matrix values range from 0 to 2. The original data present a tie between *Lathyrus pratensis* and two clusters of grasspea: one formed with *Lathyrus clymenum* and *Lathyrus ochrus* and the other with *Lathyrus sylvestris* and *Lathyrus latifolius*. The corresponding multidendrogram is shown in Figure 1A, where the minimum and maximum distances between all cluster elements are 1.0459 and 1.1112, respectively. This tie is responsible for two different binary dendrograms using the UPGMA method. We can describe this tie as happening in the middle of the dendrogram as it is formed by taxa already in clusters. After its formation, the tied cluster will be grouped with the other five elements in the dendrogram. This case clearly shows that tied distances can happen in any step of the clustering process.

In Figure 1B, we can observe one of the two possible binary dendrograms, clustering first *L. pratensis* with the pair formed by *L. sylvestris* and *L. latifolius*. Then, a second cluster formed with *L. clymenum* and *L. ochrus* is added to the previous cluster containing three elements. This binary dendrogram shown in Figure 1B is exactly the same that the authors of the study gave in their article [20]. In Figure 1C, it is depicted the other possible binary dendrogram for the same input data, where *L. clymenum* and *L. ochrus* are clustered first with *L. sylvestris* and *L. latifolius*. A fifth element, *L. pratensis*, is added then to the previous cluster containing four elements.

In the second case study, the authors analyze the genetic diversity of 22 chillies (*Capsicum annuum L.*) germplasm using four microsatellite markers [41]. The article provides a proximity matrix of similarity values with an accuracy of three decimal digits. The similarity matrix values range from 0 to 1. The original data present three ties along with the resulting multidendrogram (Figure 6A). These three ties are responsible for more than 2.5 million different binary dendrograms using the UPGMA method (to be exact, 2655193 different binary dendrograms). This second case study is a clear example that multiple ties can occur in the same hierarchical clustering. Note that the larger the data set, the more likely it is to have different binary dendrograms [18].

In Figure 6, we can also observe two possible binary dendrograms for the 22 chillies among the more than 2.5 million possibilities. The two selected dendrograms have several remarkable differences between them. One clear difference, for instance, is that in Figure 6B *Comilla* is first clustered with *Sada_gol*, and the resulting cluster is merged with *Ruma*. On the contrary, in Figure 6C *Comilla* is first clustered with *Dhani*, and the resulting cluster is merged with *Sada_gol*. An even more outstanding difference is found between clusters (*Angoor*, *Shada*) and (*Boro*, *BD.2025*) that are directly clustered together in Figure 6C, whereas they only join at the root of the dendrogram (minimum ultrametric similarity) in Figure 6B.

## Conclusions

In this study, we have provided a comprehensive review of microsatellite dendrograms created using the UPGMA method. Binary dendrograms are constrained to group elements in pairs, which becomes an issue when there are tied distances between two or more elements. In these cases, a binary dendrogram first groups two of the tied elements, and then the other elements in the tie are added in a posterior step or they are grouped in another cluster. In this way, the real genetic relationship between genotypes is not properly reflected in the hierarchical clustering. For this reason, we wanted to know how frequently ties in proximity occur in published hierarchical clusterings of microsatellite markers. Our analysis shows that 46% (95% CI 36–56%) of the articles have at least one alternative solution to the published binary dendrogram. In our dataset of 2239 articles, this would correspond to 1032 articles having at least one tie (95% CI 816–1248 articles). The potential implications that this finding uncovers need to be taken seriously into consideration because between one-third and up to one-half of the articles under consideration are affected by the ties in proximity problem. The existence of articles containing UPGMA binary dendrograms that are not unique solutions can have consequences not only on the direct conclusions obtained in these publications, but also indirectly on the works based on these original publications.

With such alarming numbers, one has to consider what a suitable solution for this problem is. The results shown in this study are a clear example of why hierarchical clustering studies should not limit their results to a single binary dendrogram when ties in proximity can cause up to hundreds or even thousands of different binary dendrograms, for a given set of genotypes. In the introduction of this manuscript, we have mentioned some possible solutions such as analyzing all the resulting binary dendrograms by counting cluster frequencies [16] or by assessing distances among elements [26]; using pyramidal clustering to allow cluster overlapping [27–29] or grouping more than two clusters at the same time using multidendrograms [30].

Ties in proximity affect more fields than the one analyzed here. We have shown that ties are not exclusive of biological sciences or similar research areas; instead, they can also occur in completely different research areas. Thus, such a wide range of research topics affected by ties is not exclusive of microsatellite data and experiments. Note that this problem is inherent in the methodology used to obtain binary hierarchical clusterings. Here we have studied the UPGMA method in particular, although the same problem can occur using any other type of agglomerative hierarchical clustering, such as the also common complete linkage or Ward methods.

**Key Points**

- Dendrograms are used in microsatellite marker publications.
- There are microsatellite distance ties in 46% of the papers.
- Several binary dendrograms are generated although not reported.
- This situation makes results not concluding nor reproducible.
- We propose to use algorithms that generate unique dendrograms.

## Supplementary Material

supplementary_data_bbac312

## Author Biographies

**Natàlia Segura-Alabart** is a PhD candidate at the Departament d’Enginyeria Informàtica i Matemàtiques of Universitat Rovira i Virgili, Tarragona (Spain). Her current research interests include bioinformatics, proteomics and machine learning.

**Francesc Serratosa** is a full professor at the Departament d’Enginyeria Informàtica i Matemàtiques of Universitat Rovira i Virgili, Tarragona (Spain). His research interests include bioinformatics, machine learning and pattern recognition.

**Sergio Gómez** is a professor at the Departament d’Enginyeria Informàtica i Matemàtiques of Universitat Rovira i Virgili, Tarragona (Spain). His research interests include bioinformatics, complex networks and machine learning.

**Alberto Fernández** is a professor at the Departament d’Enginyeria Química of Universitat Rovira i Virgili, Tarragona (Spain). His research interests include bioinformatics, machine learning and statistical inference.
